# Percutaneous mechanical atherothrombectomy versus arterial bypass surgery for femoropopliteal in-stent restenosis: a budget impact analysis

**DOI:** 10.1016/j.jscai.2025.103616

**Published:** 2025-06-17

**Authors:** Benedict Stanberry, Drew Maclean, Ahmed Elbasty

**Affiliations:** a Institute of Healthcare Leadership and Management, Oxford, United Kingdom; b Department of Interventional Radiology, University Hospital Southampton NHS Foundation Trust, Southampton, United Kingdom; c Department of Vascular and Endovascular Surgery, University Hospital Southampton NHS Foundation Trust, Southampton, United Kingdom

**Keywords:** femoropopliteal, in-stent restenosis, percutaneous mechanical atherothrombectomy, Rotarex

## Abstract

**Background:**

There is a growing body of evidence attesting to the safety and efficacy of percutaneous mechanical atherothrombectomy (PMA) for patients with femoropopliteal (FP) in-stent restenosis (ISR) and occlusion. This study aimed to compare the costs of PMA with those of arterial bypass surgery and analyze the potential impact the introduction of PMA could have on the budget of a typical vascular service.

**Methods:**

A budget impact model with a 5-year time horizon was developed assuming an annual caseload of 12 patients with FP-ISR for whom arterial bypass surgery was the only intervention available for treating FP-ISR before the introduction of a PMA device (Rotarex; BD) and that, after its introduction, 50% of patients would be treated by this endovascular approach. Interviews with local clinical experts were used to map these 2 treatment pathways in detail, and all other inputs were sourced from national registries and published literature. All costs were based on 2022 pound sterling. Uncertainty in the model was explored through one-way sensitivity analysis and through 2 alternative scenarios that investigated the impact of different FP-ISR incidence rates, intervention mixes, and postsurgery lengths of stay.

**Results:**

The analysis estimated that the introduction of PMA into future practice at a typical vascular service could achieve a cost saving of £4750 for each patient with FP-ISR undergoing an endovascular procedure instead of bypass surgery. This became a total budget impact of £142,497 over the model’s 5-year time horizon. Reductions in postsurgery lengths of stay, delayed discharges, and operating theater utilization were the major drivers of cost savings.

**Conclusions:**

The introduction of PMA as a treatment option for FP-ISR can deliver significant cost savings for vascular services due to substantial reductions in inpatient bed and operating theater use that are maintained across sensitivity and scenario analysis. It has a valuable role to play in improving day surgery rates and reducing the intensity of vascular surgery workloads.

## Introduction

Peripheral arterial disease is an atherosclerotic disease of the peripheral vasculature leading to arterial stenosis or occlusion of the lower limbs.^1^ The disease’s rising prevalence is driving an increase in the number of patients being treated for its end-stage manifestation: chronic limb-threatening ischemia.^2^ Chronic limb-threatening ischemia is generally thought to affect 1% of the population^3^^,^^4^ and known to have a very poor prognosis: 22% of patients will lose their leg within 12 months of diagnosis and another 22% will die.^5^

Given the limitations of conservative management of chronic limb-threatening ischemia, revascularization is often essential to limb preservation.^6^ Open surgical procedures can involve endarterectomy and/or arterial bypass with an autologous vein or prosthetic graft but are associated with high morbidity and mortality as well as considerable resource use.^7^ Endovascular revascularization involving percutaneous transluminal angioplasty (PTA) and/or stenting is therefore becoming more widely applied and is recommended as first-line treatment for short, focal femoropopliteal (FP) lesions.^8^ Figures from the United Kingdom’s National Vascular Registry show that 2871 endovascular procedures (vs 5387 bypass procedures) were carried out in 2014^9^ and that this had risen to 9447 procedures (vs 7114 bypass procedures) by 2023.^10^

Unfortunately, both angioplasty and stenting can damage the arterial wall and the resulting in-growth within the cylinder of a stented artery, as well as within small margins proximal and distal to the stent, will cause a loss of luminal volume.^11^ Hence, behind “the seductive angiographic appearance of the freshly stented femoral artery” lays one of the greatest challenges facing vascular patients: that of in-stent restenosis (ISR) and occlusion.^12^ Classified under a system devised by Tosaka et al,^13^ total occlusions (class III) correlate with significantly worse prognoses than focal (class I) or diffuse (class II) FP-ISR lesions.^14^ Although reported incidence varies and is likely underreported, approximately 40% of the current generation of bare-metal stents will exhibit ISR after 1 year, rising to 60% after 3 years.^15^ Drug-eluting stents can provide superior long-term patency.^16^

There are a number of possible treatment approaches for FP-ISR—including bypass surgery and PTA (with or without restenting)—but current guidelines do not offer strong recommendations, nor is there any expert consensus on the standard of care.^17^ Conventional PTA does not yield satisfactory results,^18^ and a systematic review of 4 randomized controlled trials of FP-ISR treatments has concluded that the most effective approach remains disputable.^19^ Arterial bypass surgery therefore remains a feasible and effective, although more invasive, treatment strategy that has been demonstrated to offer similar outcomes to endovascular treatments—albeit with longer associated hospital stays^20^ and higher morbidity and mortality.^7^

There is an emerging body of evidence attesting to the safety and efficacy of percutaneous mechanical atherothrombectomy (PMA) devices.^21^ They show promising results for patients with FP-ISR—particularly those with more complex and challenging lesions, whose comorbidities make them unfit for surgery or who refuse surgical reconstruction.^22^ One such device, Rotarex (BD), uses a rotating mechanism to actively modify, excise, and aspirate plaque and thrombus from peripheral arterial lesions and occlusions.^23^ It is indicated for native blood vessels, native or artificial bypasses, and vessels fitted with stents or stent grafts. Although mechanical disruption of FP-ISR lesions can generate downstream debris and potential embolic materials that require distal embolization and antirestenotic measures, atherothrombectomy devices that are indicated for use in FP-ISR have become a widely accepted treatment tool because reducing smooth muscle proliferation theoretically avoids the need for repeat revascularization and restenting.^24^ A number of studies, including nonrandomized comparisons, have shown PMA to be safe and effective in this indication.^25^, ^26^, ^27^, ^28^, ^29^

A recent multicenter study has explored the cost, from a payer’s perspective, of open surgery and endovascular procedures for symptomatic FP-ISR,^30^ but, to date, no economic evaluation has analyzed these procedures from a provider’s perspective nor done so using detailed real-world treatment pathways and microcosting techniques. The objective of this budget impact analysis (BIA) was therefore to compare the costs of PMA with the costs of arterial bypass surgery and to analyze the potential impact its introduction could have on the budget of a typical vascular service.

## Methods

Budget impact analysis is an essential part of a comprehensive economic assessment of a health care technology. It provides a framework that enables decision-makers to see how the costs and outcomes associated with the way they currently treat a specific disease or condition would change if they adopted a new treatment or intervention and the affect this would consequently have on their annual budget and resource use.^31^ Our BIA was developed in the form of an interactive cost calculator in Microsoft Excel, and a base case was built using real-world evidence (RWE) from the vascular service at University Hospital Southampton National Health Service (NHS) Foundation Trust (UHS) supplemented with data from national registries and published literature. It was designed to estimate the budget impact of introducing PMA (with adjunctive PTA and/or restenting if required) as an alternative to arterial bypass surgery for a hypothetical annual caseload of patients with FP-ISR with focal or diffuse lesions (Tosaka class I or II) or total occlusions (Tosaka class III). It was prepared in accordance with 2 sets of well-regarded standards: the Principles of Good Practice for Budget Impact Analysis published by the International Society for Pharmacoeconomics and Outcomes Research (ISPOR)^32^ and the Assessing Resource Impact Process Manual of the National Institute for Health and Care Excellence.^33^

### Perspective

Since a significant proportion of their current and future workload may arise from FP-ISR, our analysis takes a provider perspective—that is, that of a typical vascular service. It is intended to inform decision making by vascular services and specialist vascular teams by calculating the costs associated with arterial bypass surgery (which, due to the clinical team’s concerns regarding the increased risk of death associated with paclitaxel-coated balloons and stents,^34^ was the only intervention available for treating FP-ISR at UHS before the introduction of PMA) and comparing them with the costs of PMA.

### Intervention mix

Our model compares the production costs a vascular service incurs at each stage in the inpatient pathway for arterial bypass surgery under general anesthetic with those incurred at each stage in the day case pathway for endovascular recanalization performed under local anesthetic using PMA with adjunctive PTA and/or restenting where indicated.

### Population

The Hospital Episode Statistics for UHS state that 30 patients underwent percutaneous transluminal insertion of a stent into their femoral artery in 2022.^35^ Our base case therefore uses an annual caseload of 12 patients to represent a 1-year FP-ISR rate of 40%.^15^ The interactive version of our BIA prepared in Microsoft Excel enables users to enter their own caseloads. Since Hospital Episode Statistics data show neither consistent growth nor contraction in femoral stent insertion rates over the last 5 years our base case assumes there will be no change in the caseload over the time horizon of the model.

### Time horizon

This BIA has a 5-year time horizon, since this is the financial payback period most commonly used in NHS business cases. Results are presented for each annual budget period within this time horizon. As per ISPOR guidelines, no discounting was applied and no adjustments were made for inflation.^32^ All costs refer to 2022.

### Analytic framework

Our BIA was developed using time-driven activity-based costing—a bottom-up cost-accounting methodology that enables granular comparison of the costs of different treatment pathways.^36^^,^^37^ Detailed pathway mapping took place in collaboration with local clinical experts. Each pathway was defined as beginning when a symptomatic patient with FP-ISR presented at a rapid access vascular clinic and ending with the final follow-up appointment. A high-level process map (Central Illustration) was developed to illustrate the main steps in each pathway and more detailed maps developed for each step in each pathway.

**Central Illustration:**
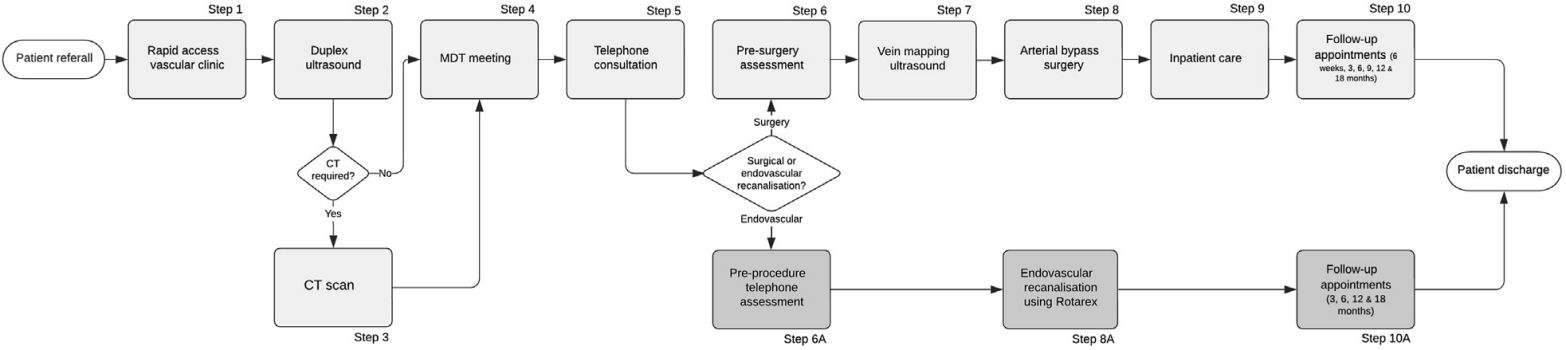
**High-level process map of FP-ISR treatment pathways at UHS.** CT, computed tomography; FP-ISR, femoropopliteal in-stent restenosis and occlusion; MDT, multidisciplinary team; UHS, University Hospital Southampton NHS Foundation Trust.

### Input data

Calculation and comparison of treatment pathway costs using time-driven activity-based costing requires estimates of the following: (1) the duration of each activity in each step of each pathway and (2) the unit costs of the resources used by the activity. Activity durations were obtained directly from the detailed process maps and multiplied by the unit cost of the personnel performing the activity. These unit costs—sourced directly from the Unit Costs of Health and Social Care Programme^38^—include total employment costs, the costs of equipment and consumables, and the costs of indirect labor, material, and capital overheads.^39^

Our base case uses UHS’s actual median length of stay (LOS) for arterial bypass surgery of 6.0 days.^10^ The 21.3% probability of delayed discharge (defined as a patient continuing to occupy a hospital bed after they are medically fit to leave hospital) and median additional LOS of 7.0 days were sourced from Houghton et al.^40^

Probabilities for adverse events occurring after surgery were obtained from a systematic review by van de Weijer et al.^41^ The additional LOS due to wound and graft infection was sourced from Totty et al^42^ and for bleeding, whether or not requiring a return to surgery, from Stokes et al.^43^ Local clinical expert opinion was used to estimate the additional resources required to manage lymphedema and seroma. The probabilities for adverse events occurring after endovascular recanalization with Rotarex were obtained from Milnerowicz et al^26^ and expert local opinion was used to estimate their impact on procedure time. The cost of a single-use catheter set (which includes a guide wire, collecting bag, and sterile drape) and drive unit was obtained from the manufacturer (BD)—the latter is assumed to be provided at no cost as part of a catheter contract deal. Since April 2016 a nationwide system has been in operation under which devices such as balloons and stents are purchased and supplied directly by NHS England at no-cost to vascular services.

### Analyses

The budget impact per patient was calculated as the incremental difference between the total costs of the surgical pathway and those of the endovascular pathway, taking into account the impact of delayed discharges and complications. The total budget impact over the 5-year time horizon was calculated using the intervention mix and population described earlier. The interactive version of the BIA also enables users to understand the number of inpatient bed days and operating theater hours that are released by the replacement of bypass surgery with endovascular recanalization.

### Uncertainty

The ISPOR guidelines do not recommend performing probabilistic sensitivity analysis on BIAs.^32^ A deterministic one-way sensitivity analysis was therefore conducted on all base case input parameters using upper and lower bounds to understand the impact of each of them on the resulting budget impact. The complication rates derived from the published literature were varied according to the same 95% CIs used in the source studies.^26^^,^^40^, ^41^, ^42^ Upper and lower bounds of 20% were used for the costs of each step in each treatment pathway with the exception of step 9 (inpatient care) for which the 6-day median postsurgery LOS published by the United Kingdom National Vascular Registry was varied according to its IQR of 2 to 12 days.^10^ The median additional LOS due to delayed discharge was also varied according its IQR of 3 to 13 days.^40^ Device costs were varied by 5%.

Our model’s base case uses default parameters and assumptions based on RWE from the vascular service at UHS. However, the interactive version prepared in Microsoft Excel enables users to enter their own input parameters in order to create scenarios that mimic their current practice or represent hypothetical future practices. For our scenario analyses, we used United Kingdom National Vascular Registry data for 2 different vascular services that perform lower limb revascularization procedures.^10^ For scenario 1, we used a vascular service with a bypass-first orientation, a short postsurgery LOS and low FP-ISR rates—that is, with characteristics that would minimize the impact of PMA. Scenario 2 estimates the device’s impact on a service with characteristics that would maximize its impact—namely a best endovascular treatment first orientation, long postsurgery LOS, and high FP-ISR rates.

## Results

### Treatment pathway costs

Table 1 shows the results of the analysis of the costs of the 2 treatment pathways. The first 5 steps are common to both pathways and calculated to cost £369.^26^^,^^41^, ^42^, ^43^ Thereafter, arterial bypass surgery requires a further 5 unique steps and costs an additional £6921. In this pathway, a day of inpatient care costs £628, and the surgical procedure itself costs £2708. Delayed discharges add £937 to the surgical pathway cost, and complications contribute a further £1700, for a total cost of £9927.

**Table 1:** FP-ISR treatment pathway cost analysis.

Input parameter	Arterial bypass surgery				Endovascular recanalization with PMA				Source(s)
	Cost, £	Probability, %	Utilization	Total, £	Cost, £	Probability, %	Utilization	Total, £	
Step 1: Rapid access vascular clinic	88	100	1.0	88	88	100	1.0	88	Real-world evidence gathered from multiple interviews with local clinical experts
Step 2: Duplex ultrasound	48	100	1.0	48	48	100	1.0	48	
Step 3: CT scan	85	50	1.0	42	85	50	1.0	42	
Step 4: MDT meeting	173	100	1.0	173	173	100	1.0	173	
Step 5: Telephone consultation	17	100	1.0	17	17	100	1.0	17	
Step 6: Presurgery assessment	108	100	1.0	108	–	–	–	–	
Step 7: Vein-mapping ultrasound	48	100	1.0	48	–	–	–	–	
Step 8: Arterial bypass surgery	2708	100	1.0	2708	–	–	–	–	
Step 9: Inpatient care (per day)	628	100	6.0	3770	–	–	–	–	
Step 10: Surgical follow-up appointments	48	100	6.0	287	–	–	–	–	
Step 6A: Preprocedural telephone assessment	–	–	–	–	17	100	1.0	17	
Step 8A: Endovascular recanalization procedure	–	–	–	–	878	100	1.0	878	
Step 10A: Endovascular follow-up appointments	–	–	–	–	48	100	1.0	192	
Catheter set	–	–	–	–	3600	100	1.0	3600	BD
Delayed discharge	628	21.3	7.0 d	937	–	–	–	–	Houghton et al,^40^ 2016
Surgical complications (<30 d)									
Wound infection	628	7.4	7.5 d	350	–	–	–	–	Probabilities: van de Weijer et al,^41^ 2015; LOS: Totty et al,^42^ 2021
Wound infection with necrosis	628	0.4	7.5 d	24	–	–	–	–	
Graft infection	628	2.4	7.5 d	113	–	–	–	–	
Bleeding (without return to surgery)	628	4.9	9.3 d	286	–	–	–	–	Probabilities: van de Weijer et al,^41^ 2015; LOS: Stokes et al,^43^ 2011
Bleeding (requiring return to surgery)	1982	2.5	9.3 d	495	–	–	–	–	
Occlusion (requiring repeat procedure)	3336	12.0	5.3 d	400	–	–	–	–	
Lymphedema	628	2.9	1.0 d	18	–	–	–	–	Probabilities: van de Weijer at al,^41^ 2015; LOS: expert opinion
Seroma	628	2.0	1.0 d	13	–	–	–	–	
PMA complications									
Embolization (resolved by tPA)	–	–	–	–	73	2.7	10 mins	2	Probabilities: Milnerowicz et al,^26^ 2019; LOS: expert opinion
Embolization (resolved by tPA+DES)	–	–	–	–	460	5.5	20 mins	25	
Dissection (no additional treatment required)	–	–	–	–	0	4.1	0 mins	0	
Perforation (requiring implantation of stent-graft)	–	–	–	–	73	1.4	10 mins	1	
Uncrossable occlusion (requiring reintervention)	–	–	–	–	6634	1.4	6.0 days	93	
Total treatment pathway cost, £				9927	–	–	–	5177	
Incremental difference in treatment pathway cost, £				–	–	–	–	4750	

CT, computed tomography; DES, drug-eluting stent; FP-ISR, femoropopliteal in-stent restenosis; LOS, length of stay; MDT, multidisciplinary team; PMA, percutaneous mechanical atherothrombectomy; tPA, tissue plasminogen activator.

All endovascular recanalization procedures are undertaken as day cases at UHS so do not carry the risk of delayed discharge. The procedure itself takes place in a catheterization laboratory rather than an operating room, and there are fewer follow-up appointments than after surgery. Complications, which with 1 exception are resolvable during the procedure, add an additional £121, for a total cost of £5177.

Many steps in the surgical pathway are either less costly in the endovascular pathway or are eliminated entirely—including preprocedural assessment (£17 vs £108), the procedure itself (£878 vs £2708), inpatient care (£0 vs £3770), delayed discharges (£0 vs £937), complications (£121 vs £1700) and follow-up appointments (£192 vs £287). Endovascular recanalization with PMA becomes more costly than bypass surgery (£4478 vs £2708) if the cost of the catheter set is included in the procedure costs. However, the full endovascular pathway, nonetheless, results in an overall cost saving of £4750 compared with that of the surgical pathway.

### Base case analysis

Table 2 shows the results of the fully developed base case analysis in which there is a stable pool of 12 patients with FP-ISR in each budget period over the 5-year time horizon. When surgery is the only intervention available in current practice, the vascular service’s total treatment costs are £595,608. But, in a future practice in which half of these patients undergo PMA, with adjunctive PTA and restenting if required, these costs are reduced to £453,111—delivering a total cost saving of £142,497.

**Table 2:** Base case results.

	Year 1	Year 2	Year 3	Year 4	Year 5	Total
Percutaneous transluminal stent insertion procedures	30	30	30	30	30	150
TLR procedures for FP-ISR, %	12 (40)	12 (40)	12 (40)	12 (40)	12 (40)	60 (40)
Arterial bypass surgery, %						
Current practice	12 (100)	12 (100)	12 (100)	12 (100)	12 (100)	60 (100)
Future practice	6 (50)	6 (50)	6 (50)	6 (50)	6 (50)	30 (50)
Endovascular recanalization with PMA, %						
Current practice	0 (0)	0 (0)	0 (0)	0 (0)	0 (0)	0 (0)
Future practice	6 (50)	6 (50)	6 (50)	6 (50)	6 (50)	30 (50)
Step 1: Rapid access vascular clinic, £						
Current practice	1062	1062	1062	1062	1062	5308
Future practice	1062	1062	1062	1062	1062	5308
Step 2: Duplex ultrasound, £						
Current practice	575	575	575	575	575	2874
Future practice	575	575	575	575	575	2874
Step 3: CT scan, £						
Current practice	507	507	507	507	507	2535
Future practice	507	507	507	507	507	2535
Step 4: MDT meeting, £						
Current practice	2077	2077	2077	2077	2077	10,384
Future practice	2077	2077	2077	2077	2077	10,384
Step 5: Telephone consultation, £						
Current practice	208	208	208	208	208	1,038
Future practice	208	208	208	208	208	1,038
Step 6: Presurgery assessment, £						
Current practice	1301	1301	1301	1301	1301	6504
Future practice	650	650	650	650	650	3252
Step 7: Vein-mapping ultrasound, £						
Current practice	575	575	575	575	575	2874
Future practice	287	287	287	287	287	1437
Step 8: Arterial bypass surgery, £						
Current practice	32,491	32,491	32,491	32,491	32,491	162,454
Future practice	16,245	16,245	16,245	16,245	16,245	81,227
Step 9: Inpatient care, £						
Current practice	45,242	45,242	45,242	45,242	45,242	226,211
Future practice	22,621	22,621	22,621	22,621	22,621	113,105
Step 10: Surgical follow-up appointments, £						
Current practice	3448	3448	3448	3448	3448	17,241
Future practice	1724	1724	1724	1724	1724	8621
Step 6A: Preprocedural telephone assessment, £						
Current practice	0	0	0	0	0	0
Future practice	104	104	104	104	104	519
Step 8A: Endovascular recanalization procedure using PMA, £						
Current practice	0	0	0	0	0	0
Future practice	5267	5267	5267	5267	5267	26,337
Step 10A: Endovascular follow-up appointments, £						
Current practice	0	0	0	0	0	0
Future practice	1149	1149	1149	1149	1149	5747
Catheter sets, £						
Current practice	0	0	0	0	0	0
Future practice	21,600	21,600	21,600	21,600	21,600	108,000
Delayed discharges, £						
Current practice	11,243	11,243	11,243	11,243	11,243	56,213
Future practice	5621	5621	5621	5621	5621	28,107
Complications (<30 d), £						
Current practice	20,394	20,394	20,394	20,394	20,394	101,972
Future practice	10,197	10,197	10,197	10,197	10,197	50,986
Total treatment cost, £						
Current practice	119,122	119,122	119,122	119,122	119,122	595,608
Future practice	90,622	90,622	90,622	90,622	90,622	453,111
Net budget impact (incremental difference in treatment cost), £						
Current practice	–	–	–	–	–	–
Future practice	28,499	28,499	28,499	28,499	28,499	142,497

CT, computed tomography; FP-ISR, femoropopliteal in-stent restenosis; MDT, multidisciplinary team; PMA, percutaneous mechanical atherothrombectomy; TLR, target lesion revascularization.

### One-way sensitivity analysis

**Figure 1:**
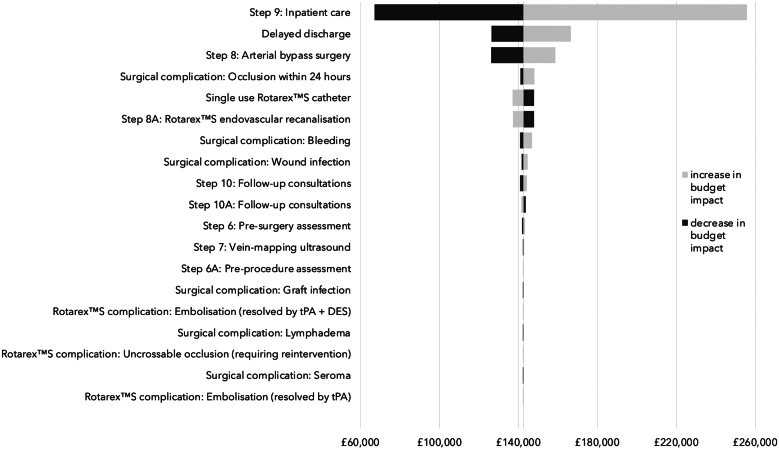
**One-way sensitivity analysis of base case results.** DES, drug-eluting stent, tPA, tissue plasminogen activator.

The results of the one-way sensitivity analysis of the base case input parameters are illustrated as a Tornado diagram in Figure 1. They demonstrate that the majority of the cost savings available to a vascular service from introducing PMA to treat patients with FP-ISR arise from the elimination of inpatient stays (lower bound: £67,093; upper bound: £255,602). Significant additional savings are achieved by avoiding delayed discharges (lower bound: £126,436; upper bound: £166,588) and from the release of operating theater capacity (lower bound: £126,251; upper bound: £158,742). The cost savings are also somewhat influenced by reductions in the more serious surgical complications such as occlusion (lower bound: £141,055; upper bound: £148,141), bleeding (lower bound: £140,763; upper bound: £146,855) and wound infection (lower bound: £141,621; upper bound: £144,696). Overall, the results of the sensitivity analysis show that the estimated cost savings are highly robust and that, even if a vascular service has a very low median postsurgery LOS of 2.0 days, PMA will still have a beneficial budget impact.

### Scenario analysis

The results of the scenario analyses in Table 3 confirm how persistently PMA delivers cost savings for vascular services. The service in scenario 1 has a bypass-first orientation, short median postsurgery LOS and low 1-year FP-ISR incidence rate but still achieves a modest cost saving of £13,630. In scenario 2, a service with a best endovascular treatment first orientation, lengthy median postsurgery LOS and high 1-year FP-ISR incidence rate receives a substantial cost saving of £654,253 over the 5-year time horizon of the model. In this latter scenario, there are also significant service capacity benefits: 1160 inpatient bed days and 311 operating theater hours are released.

**Table 3:** Results of the scenario analyses.

Analysis	Femoral artery stent insertions (per year)	12-mo FP-ISR incidence rate, %	TLR procedures (per year)^a^	Current practice		Future practice		Median (IQR) postsurgery LOS, d	Results (over 5-y time horizon)		
				Arterial bypass surgery, %	Endovascular recanalization with PMA, %	Arterial bypass surgery, %	Endovascular recanalization with PMA, %		Total budget impact, £	Inpatient bed days released	Operating theater hours released
Base case	30	40	12	100	0	50	50	6 (2-12)	142,497	273	130
Scenario 1^a^	30	20	6	100	0	80	20	2 (2-5)	13,630	31	26
Scenario 2^b^	30	60	18	100	0	20	80	13 (6-17)	654,253	1160	311

FP-ISR, femoropopliteal in-stent restenosis; LOS, length of stay; PMA, percutaneous mechanical atherothrombectomy; TLR, target lesion revascularization.

a Deviates from the base case by assuming a lower FP-ISR incidence rate leading to fewer TLR procedures, a bypass-first orientation and a low median post-bypass LOS.

b Deviates from the base case by assuming a higher FP-ISR incidence rate leading to more TLR procedures, a best endovascular treatment first orientation and a high median post-bypass LOS.

## Discussion

This is the first BIA to explore the cost impact, from a provider’s perspective, of adopting PMA with adjunctive PTA, with or without repeat stenting, as an alternative to arterial bypass surgery for the treatment of FP-ISR. Our analysis has used RWE from a typical UK vascular service, supplemented with data from national registries and published literature, to demonstrate the device’s potential to deliver significant cost savings. These cost savings are maintained across sensitivity and scenario analysis, confirming the robustness of these results even when conservative input parameters are assumed. The endovascular approach has, furthermore, demonstrable potential to increase a vascular service’s inpatient bed and operating theater capacity by improving day surgery rates.

FP-ISR remains a pervasive challenge despite a plethora of competing treatments. Up to one-half of all patients that receive FP stents will require secondary interventions due to ISR.^12^ Consqeuently, there remains an unmet need for more effective treatments. Published results of studies of PMA devices show that—whether used alone or in combination with PTA—they consistently deliver excellent primary patency rates compared with those of other endovascular treatment options for FP-ISR.^44^ Moreover, several systematic reviews and meta-analyses have shown that percutaneous debulking devices such as this deliver more promising and favorable results than PTA or drug-coated balloon alone—particularly for patients with longer, more complex lesions.^21^^,^^45^ Atherthrombectomy systems, combined with antirestenotic measures, are therefore becoming the favored treatment strategy for such lesions.

Day surgery has an important strategic role to play for both health care payers and providers who want to take a more value-based approach to vascular services.^46^ However, despite it being estimated that day surgery costs are 25% to 68% lower than those for inpatient surgery,^47^ there remains wide variation in day case rates.^48^ Our BIA has used RWE to demonstrate that, when combined with a well-designed day surgery pathway, PMA can provide significant cost savings and release valuable hospital resources. They are of particular importance given that increasing demand for vascular care is occurring at a time when the vascular surgery workforce is shrinking and experienced vascular surgeons are being lost to retirement and burnout.^49^, ^50^, ^51^ There is therefore a pressing need for endovascular innovations capable of enhancing the productivity of vascular services and mitigating the intensity of vascular surgery workloads.

### Study limitations

Direct observation would have been the most rigorous method by which to map treatment pathways and measure activity durations. However, resource limitations and access constraints necessitated the use of staff interviews to obtain these data. Recall bias was minimized by interviewing multiple local clinical experts to obtain uniform time estimates.

Checking annual procedure volumes for femoral stent insertion at NHS vascular services was straightforward because this activity is routinely recorded in Hospital Episode Statistics data for admitted patient care under its ICD-10 code (L63.5). However, neither the code series for complications of vascular prosthetic devices, implants, and grafts (T82) nor the specific code for stenosis of a peripheral vascular stent (T82.856) are recorded in Hospital Episode Statistics data. Consequently, our use of FP-ISR rates from published studies—most of which were randomized controlled trials—may not be perfectly indicative of real-world prevalence.

Our BIA has considered a specific patient population: those requiring treatment for symptomatic FP-ISR. However, by far the largest patient populations that can benefit from atherothrombectomy, whether as a standalone or a combination treatment, are those with acute limb ischemia and chronic limb-threatening ischemia. Future economic evaluations should therefore analyze the impact of PMA on the costs of treating these important indications, from both provider and payer perspectives.

This BIA is based upon RWE from the UHS vascular service. Since FP-ISR treatment pathways, clinical practices and unit costs can vary from hospital to hospital and from country to country, the results obtained from this analysis may not be predictive for all services. Some health care systems may not fully fund or reimburse the cost of adjunctive devices, such as balloons and stents, which are used in combination with PMA.

PMA is a specific endovascular approach that uses a device that some vascular service providers may perceive as costly. In the future a comparison of the long-term costs and consequences of treating FP-ISR with a percutaneous debulking device and adjunctive PTA/drug-coated balloon versus treatment with PTA/drug-coated balloon alone is required to identify the most cost-effective treatment strategy.

## Conclusion

The introduction of PMA as a treatment option for FP-ISR can deliver significant cost savings for vascular services due to substantial reductions in inpatient bed and operating theater use that are maintained across sensitivity and scenario analysis. It has a valuable role to play in improving day surgery rates and reducing the intensity of vascular surgery workloads.
